# Is Job Insecurity Harmful to All Types of Proactivity? The Moderating Role of Future Work Self Salience and Socioeconomic Status

**DOI:** 10.3389/fpsyg.2022.839497

**Published:** 2022-02-24

**Authors:** Kaiyuan He, Jigan Wang, Muyun Sun

**Affiliations:** ^1^Business School, Hohai University, Nanjing, China; ^2^Research Institute of Human Resources, Ministry of Water Resources, Hohai University, Nanjing, China; ^3^School of Marxism, Nanjing University of Aeronautics and Astronautics, Nanjing, China

**Keywords:** job insecurity, proactive behavior, future work self salience, socioeconomic status, self-control

## Abstract

How and when do uncertain factors affect employees’ different types of proactive behavior? Building on the strength model of self-control, the present study examines the different effects of job insecurity on individual-oriented and organizational-oriented proactive behaviors, and the moderating role of future work self salience (FWSS) and socioeconomic status (SES). Two-wave data collected from 227 employees in China were used to test our hypotheses. The results indicate that job insecurity is negatively associated with all the proactive behaviors. Moreover, the FWSS positively moderates the above relationship, and the moderating role on individual-oriented proactive behavior is stronger than organizational-oriented proactive behavior. The SES negatively moderates the relationship between job insecurity and the two types of proactive behaviors. In addition, the FWSS and SES have a three-way interactive effect on the relationship between job insecurity and individual task proactive behavior. The practical implications of these results are discussed.

## Introduction

With a more intense hit of rapidly changing environment and unpredictable factors (i.e., COVID-19), employees are increasingly expected to exhibit proactivity for the sake of both individual and organizational competitiveness (Parker et al., 2019). In the workplace, proactive behavior has been used to describe an employee’s self-initiated action that aims to change and improve the situation or oneself (Crant, 2000; Parker et al., 2006; Bindl and Parker, 2017). By definition, the purposes of such behavior involve the anticipation as well as thinking about the environment to “make things happen” (Parker et al., 2010). Recently, research has also widely demonstrated the beneficial effects of proactive behavior for a variety of positive outcomes, such as career success and organizational effectiveness (Griffin et al., 2007; Cangiano et al., 2018).

Although most scholars have considered proactive behavior as one of the indispensable factors for organizational and individual success in uncertain environments (Molina and O’Shea, 2020), the analysis of the relationship between uncertainty and proactive behavior has been more mixed and controversial (Cai et al., 2019). Some results indicated that stress and negative feelings triggered by uncertainty (i.e., job insecurity) may reduce employees’ proactivity due to the loss of resources and psychological needs (Van den Broeck et al., 2014; Jiang and Lavaysse, 2018; Yao et al., 2021). Instead, the competitive views stated that “negative social contextual factors might not always be detrimental” for proactivity (Cai et al., 2019) since the discrepancies from the current and reference values created by dissatisfaction with the *status quo* would motivate proactivity based on employees’ expectations of maintaining conformity to the desired goals (Carver and Scheier, 1982; Strauss and Parker, 2018). Therefore, these inconsistent findings suggest the need to delve more deeply into how and when uncertain factors affect employees’ proactive behavior.

In addition, little attention has been paid to the comparison of different types of proactive behavior in an uncertain context. Despite the high degree of similarity in antecedents, the fact is that the effects of negative context on different types of proactive behavior are also still unclear. For instance, it has been found that job insecurity can be detrimental to organizational-oriented proactive behavior (De Spiegelaere et al., 2014; Wang et al., 2019), but there is no direct evidence for its correlation with other studies (Niesen et al., 2017; Selenko et al., 2017; Jiang, 2018). Similarly, mixed results also exist in the relationship between job insecurity and proactive behaviors which aim at employees’ personal goals and interests. Taken together, these findings cast the strong interests of scholars with regard to the differences and undiscovered boundary conditions in the relationship between uncertainty or threat and employee proactivity.

In response to this call, the present study seeks to draw upon the strength model of self-control to examine whether or not job insecurity is harmful to different types of proactive behaviors. With the aggravation of force majeure factors, job insecurity has inevitably become one of the most threatening stressors for employees. Moreover, job insecurity, as a great source of uncertainty, has been found to be harmful to a variety of outcomes, such as negative emotions and strain (Lee et al., 2018). According to the strength model of self-control, those outcomes can lead to a state of depletion of self-control resources that undermines the proactive behavior, since such resources are also a prerequisite for employee proactivity (Muraven and Slessareva, 2003; Parker et al., 2019). Nevertheless, the model further states that if an individual is sufficiently motivated and pays more attention to the goal, then he/she would be able to overcome the depletion effects of self-control (Vohs et al., 2012). Thus, we extend this argument to job insecurity literature and suggest that although both coping with job insecurity and acting in proactivity are resource-intensive, sufficient proactive motivation and attention may be beneficial to mitigate the negative effects of job insecurity on proactive behavior. In addition, these effects may have differential outcomes for different-oriented proactive behaviors due to the specific costs and functions.

**FIGURE 1:**
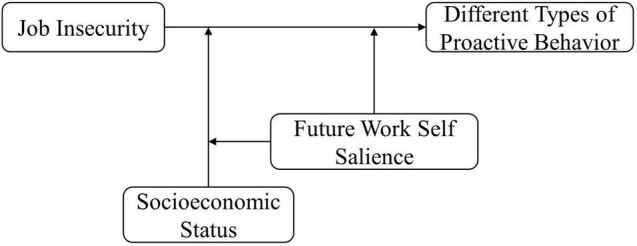
Theoretical model.

Consistent with this perspective, we further examine the moderating role of future work self salience (FWSS) (motivational factor) and socioeconomic status (SES) (contextual factor) on the link between job insecurity and different-oriented proactive behavior. First, we argue that FWSS, as a critical motivational resource for career proactivity (Strauss et al., 2012), can inhibit the result of self-control failures in the proactive behavior caused by job insecurity. Because for employees with high FWSS, the discrepancies between the current state and hoped for future not only can promote employees’ awareness of the importance and meanings of proactive behavior, but also may enable them to keep identity-congruent behaviors. Moreover, SES represents the actual or perceived amount of specific social resources. The employees with low SES, due to their lack of opportunities and resources, tend to protect available resources and act in line with the expectations of others. In contrast, individuals with high SES who experience a higher need for self-evaluation and personal control might pay more attention to the utilization of their own superior resources. It may enable the employees to exhibit different self-control strategies when confronted with the threat of job insecurity. Finally, we believe that FWSS and SES would play a three-way moderating role in the relationship between job insecurity and proactive behavior since both these two factors can affect the individual’s self-regulation and self-control. Figure 1 depicts our theoretical model.

Our investigation contributes to the literature in several ways. First, we reiterate the resource-intensive and complex nature of employee proactive behavior, and based on the strength model of self-control, we argue that there is a differential effect for uncertainty situations (e.g., job insecurity) on different types of proactive behaviors. Thus, our study advances the explanation of the mixed findings between job insecurity and proactive behavior. Second, we examine the moderating role of FWSS and SES in the effect of job insecurity on different types of proactive behaviors, respectively. We propose that FWSS and SES can facilitate the employees to overcome the effects of depletion of self-control resources, thus alleviating the failure of self-control in proactive behavior after experiencing job insecurity. Our study separately answers the question of how psychological and environmental factors mitigate the harmful effects of job insecurity on different types of proactive behaviors. Finally, our investigation also examines the three-way interactive effect of motivation and contextual factors. We advance the potential interaction effect of motivation and context on the detrimental effects of job insecurity on employee proactivity and argue that these two factors have a compensatory effect on the individuals’ ability to overcome self-control failures. This provides another perspective and empirical evidence for the development of the strength model of self-control.

## Theory Background and Hypotheses Development

### Proactive Behavior

Proactive behavior refers to organizational members’ self-initiated change to bring about a different future (Grant and Ashford, 2008). Seen from the perspective of self-control theory, the nature of proactive behavior captures the process in which individuals reduce the discrepancies between the current and potential reference values through goal setting and goal pursuing (Bindl and Parker, 2010; Strauss and Parker, 2018). Theoretically, in order to successfully approach proactive goals, a self-monitoring process with circular feedback is required as follows: (1) perceiving the current state of self and situation; (2) comparing the current state with the desired reference value (e.g., the ideal state for personal career development and task achievement); and (3) if discrepancies exist, take action to reduce them (Carver et al., 2000). Hence, proactive behavior is deemed to be resource-intensive and requires a high expenditure of self-control efforts (Parker et al., 2019). More precisely, whether anticipating, planning, giving feedback, or actively coping with potential emotions and interpersonal risks, all require the individuals to invoke higher-order cognitive mental functions to control actions, integrate acts, and events across time deliberately and meaningfully (Strauss et al., 2017). It is sometimes the case that proactivity can even ultimately be depleting. Consequently, this leads scholars to contend that proactive behavior is not a specific behavior, but any process by which an individual changes himself or herself or the environment for the sake of reducing the discrepancies between the current and reference values.

Although proactive behavior is a complex phenomenon with a wealth of manifestations, numerous articles still theoretically and statistically treat proactive behavior as a unidimensional variable or directly analyze some specific proactive behavior without sufficient comparisons. In offering insight into the issue, scholars have integrated multiple types of proactive behaviors, and several taxonomies have been developed. For example, based on the behavioral target, Griffin et al. (2007) distinguished proactive behaviors into the task, team, and organization three dimensions. Furthermore, Strauss and Parker (2018) classified the above into present-oriented and future-oriented proactive behaviors based on the temporal orientation of the focused reference values in self-control when comparing the intervention conditions. In addition, scholars have recently begun to focus on proactive behaviors based on individual-oriented and organization-oriented classification perspectives. Concepts such as self-oriented job crafting (Niessen et al., 2016), and self-interested voice (Duan et al., 2021) have been proposed successively. It is necessary and required to compare proactive behaviors from this perspective. Because, by definition, proactive behaviors are not exclusively characterized as pro-organization. Moreover, based on the existing arguments, these two types of proactive behavior may bring different benefits and risks, especially in an uncertain context (Bolino and Grant, 2016). Specifically, individual-oriented proactive behaviors are more conducive to achieving employee achievements, career development, and task performance related to personal interests; while proactive behaviors that aim to improve organizational functioning would be beneficial to achieving long-term goals, but may be accompanied by higher interpersonal risk (Griffin et al., 2007). Accordingly, following Strauss and Parker’s prior work, the present study mainly focuses on comparing how negative context affects three specific proactive behaviors, namely, proactive skill development, individual task proactivity, and organizational member proactivity, which focus on skill development, task performance, and organizational development, respectively.

### Job Insecurity and Different Types of Proactive Behaviors

Job insecurity is defined as “the anticipation of this stressful event in such a way that the nature and continued existence of one’s job are perceived to be at risk” (Sverke et al., 2002). As a known unfavorable subjective experience, it occurs when an employee anticipates and forecasts a loss event about future employment, rather than the necessarily actual job loss. With reference to the individual- and work-related outcomes, a growing body of evidence has linked such perceived instability of one’s job to low level of work engagement (Park and Ono, 2017), poor psychological health (Vander Elst et al., 2014), and increasing anxiety (Cheung et al., 2019). Thus, for employees with high levels of job insecurity, threat and uncertainty are the fundamental components of their perception of the environment (Wang et al., 2015; Shoss, 2017).

We argue that in general, job insecurity undermines proactive behavior. As previously mentioned, proactive behavior requires a significant effort in self-control. However, the strength model of self-control states that self-control is an individual’s finite psychological resource and all acts that need self-control effort (e.g., pursuing future-oriented aims) are drawn from a common yet finite pool of resources (Baumeister et al., 1998; Gino et al., 2011). Therefore, self-control efforts in the early stage may result in failure later. Notably, there is compelling evidence for the fact that negative emotions, uncertain contexts, and the threat of potential resource loss can deplete an individuals’ self-control resources (Baumeister and Vohs, 2007, 2016). Unfortunately, job insecurity is the very factor that engenders negative emotions (e.g., anxiety) and threats basic psychological needs which generate a lack of control over their resources and environment. Based on this logic, coping with the above events requires a great deal of cognitive and emotional effort on the part of the individual, which may result in a failure of self-control in proactive behaviors that require the same resources. In addition, the context of job insecurity also causes a proactive behavior dilemma (Wang et al., 2015), which requires employees to judge and choose between the potential benefits and threats. In sum, the consumption of these resources is more likely to lead employees to the inability of proactivity feedback loops and ultimately to a failure of self-control in proactive behavior.

Nevertheless, the relationship between job insecurity and different-oriented proactive behaviors in empirical results is still mixed and lacks a comparative analysis. In the present study, we argue that job insecurity may be more destructive to organization-oriented proactive behaviors than individual-oriented. On the one hand, individual-oriented proactive behaviors are more likely to be “job preservation strategies” to deal with job insecurity (Shoss, 2017; Wang et al., 2018). Since in an uncertain environment, proactivity aimed at career development, and task performance can not only facilitate employees to perceive a greater sense of competence and control over their environment but is also helpful to demonstrate their self-worth to the organization. On the other hand, individual-oriented proactive behavior may result in less psychological stress and interpersonal risk than organizational-oriented proactive behavior. For employees who experience a higher degree of job insecurity, they are more likely to engage in behaviors that reduce threat due to a higher propensity for risk avoidance. Some supportive evidence suggests that job insecurity may increase facades of conformity with the organization (Hewlin et al., 2016), positively correlate job crafting in the task, and cognitive dimensions under some conditions (Buonocore et al., 2020). Therefore, we propose the following hypothesis:

*Hypothesis 1a:* The degree to which an employee perceives job insecurity will have a negative relationship with different-oriented proactive behavior.
*Hypothesis 1b:* The degree to which an employee perceives job insecurity will have a stronger negative relationship with the organizational member proactivity than the proactive skill development and individual task proactivity.

### Moderating Role of Future Work Self Salience: Motivation Factor

So far, although great progress has been made in identifying factors that can mitigate the detrimental effect of job insecurity, such as work environment and individual characteristics (Lee et al., 2018), little attention has been devoted to the buffer function of motivation. The strength model of self-control elucidates that self-control failure may be just the result of partly depleted resources during the early effort, rather than complete incapacity (Baumeister and Vohs, 2007). Motivations or perceptions of the importance of subsequent behaviors can inspire a person to expend remaining resources and overcome the conservation effect (Baumeister and Vohs, 2016). Several studies and meta-analyses have supported this notion (Boucher and Kofos, 2012; de Ridder et al., 2012; Vohs et al., 2012). Therefore, we indicated that motivation of proactivity can be particularly useful in lowering the inhibitions of individuals with higher job insecurity to proactive behavior.

Future work self salience refers to the degree to which the mental clarity and accessibility of one’s possible self that reflects his or her hopes and aspirations for future work life (Strauss et al., 2012). More importantly, researchers have indicated that FWSS captures the state such that the possible self is continuously activated and frequently used in the self-concept system, or in the chronic accessibility (Higgins, 1996; Strauss et al., 2012). Thus, from the perspective of self-control, the FWSS is served as an important motivational resource, since it is conducive for individuals to convert a distant-imaged picture of future work life to more proximal and immediate self-set goals. Then, the identification of discrepancies between the current state and for the future will motivate and enable individuals to goal decomposition and goal pursuing. These findings revealed that FWSS is positively related to career adaptation, proactive career behaviors, and work engagement (Guan et al., 2014; Taber and Blankemeyer, 2015). In this context, we argue that FWSS is a significant moderator that can mitigate the negative effect of job insecurity on proactive behavior for several reasons.

First, previous findings suggested that FWSS has a positive effect on an individual’s belief and commitment toward goal accomplishment by identifying the discrepancies between the current and projected goals and the anticipated outcomes (Strauss et al., 2012). More specifically, when the employees with more salient future work self, they not only experience a closer connection between their current behavior and the desired ideal state but also recognize the stronger importance of the current behavior. Therefore, such future-based cognition can help individuals effectively deal with the dilemma of proactive behavior in experiencing job insecurity, reducing the anxiety about whether they can ultimately reach the goal of job preservation. In other words, FWSS can serve as a “compass” that directs individuals to consistently focus and invest resources in behaviors that help them achieve their future career goals, such as accumulating experience and learning skills in their current job, while reducing their concerns about external threats. Evidence suggests when individuals perceive more importance of subsequent behavior, they will increase their willingness to consume the limited self-control resources even if it costs high (Graham et al., 2014).

Second, identity-based motivation theory assumes that individuals are motivated to act, interpret situations, and make sense of their own existent meanings in ways that are congruent with their activated identity (Oyserman, 2009). When individuals act in an identity-congruent way, even when encountering obstacles, their behaviors are more likely to be interpreted as important and meaningful rather than impossible or unnecessary (Oyserman and Destin, 2010). However, researchers based on this model suggest that not all future identities are capable of motivating an individual’s behavior, unless one considers certain future identities to be important, vivid, and continuous with the current self (Ayduk and Kross, 2010; Van Gelder et al., 2015; Nurra and Oyserman, 2018). As aforementioned, the FWSS reflects an employee’s positive and activated future career identity and expectations for constructing a relevant identity. Thus, when employees with high FWSS are confronted with job insecurity, employees might be more likely to view such stressors as meaningful barriers that must be crossed. Conversely, lower FWSS might lead employees to believe that future-focused proactive behaviors are irrelevant to their current behaviors, which further increases the perception of uncertainty and anxiety generated by the risk of potential unemployment and reduces the interpretation and perceived importance of the meaning of proactive behaviors.

Thus, in consistency with Hypothesis 1, this study assumes that the moderating role of FWSS on the relationship between job insecurity and individual-oriented proactive behavior may be stronger than the organizational-oriented proactive behavior. On the one hand, individual-oriented proactive behaviors are more likely to be consistent with the goals, values, and meanings the employees set on their own, based on their future work in the long term. On the other hand, work-related skill development and efficient task accomplishment are more beneficial to others’ recognition of employees’ value and competence, thus achieving direct control over the environment and reducing the risk of resource loss. Thus, we propose the following hypothesis:

*Hypothesis 2a:* Future work self salience will have a positively moderating role on the relationship between job insecurity and proactive behaviors. Specifically, the negative relationship between job insecurity and proactive behaviors will be weaker (vs. stronger) at high (vs. low) FWSS.
*Hypothesis 2b:* The moderating role of FWSS on the relationship between job insecurity and proactive skill development, individual task proactivity will be stronger than organizational member proactivity.

### Moderating Role of Socioeconomic Status: Contextual Factor

Our model further suggests that the SES will affect the pattern of proactivity in the context of job insecurity. The SES is typically conceptualized as an individual advantage within a society relative to others (Kraus et al., 2009). Recently, a growing literature has shown that SES, as a contextual factor, can shape individuals’ perception of self and the external world, provide a framework for how people think, act, and interact with each other. Those individuals with high SES, better resources, and more opportunities for social participation are more motivated to engage in behaviors consistent with personal needs and interests in the expectation of personal control (Laurin et al., 2011; Flores et al., 2017). Individuals with low SES tend to highly pursue goals consistent with the expectations of others due to the vulnerability to external threats and the lack of resources that can buffer against the devastating effects of an uncertain environment (Schaerer et al., 2021). Therefore, in an environment full of frustrations and challenges, the SES may enable employees to exhibit different strategies for self-control (Laurin et al., 2011). At present, whether with high or low SES, people may be at the risk of losing their jobs due to multiple factors. Given this, we argue that SES is an important contextual factor that moderates the relationship between job insecurity and proactive behavior.

Although job insecurity may motivate employees to protect their jobs (Shoss, 2017), the important precondition is grounded in the rationale that protecting rather than leaving their existing jobs is a better choice. Unlike other stressors, once the risk of unemployment becomes a materialization, it may lead to more immediate and destructive results on the lives if necessary material resources lack. Therefore, for employees with high SES, protecting their current job may not be the optimal decision, since job insecurity may cause them to perceive a stronger denial of self-worth and a disruption of psychological needs and well-being due to the higher need for personal control. In addition, more job opportunities or higher employability will lead them to focus their self-control effort on searching for other best options from a deeper “pool of opportunities” that match their abilities and career development, in order to maintain their long-term advantage (Flores et al., 2017). In contrast, low SES that represents a strong situation might require an employee to meet the employers’ expectations to keep up their job and avoid the loss of resources in the short-term due to lack of resources and higher economic vulnerability. In other words, individuals with low SES are more sensitive to threats, are more likely to apply their remaining self-control effort to avoid job loss and the following loss of resources by demonstrating self-worth and accumulating experience in an organization (Fischmann et al., 2018). Therefore, improving job skills and task performance may become a way of coping with this threatening environment. Several existing findings partially unpack the above argument. For example, Berntson et al. (2010) theorized and found that among individuals who have higher external employability experience, job insecurity will be more likely to reduce voice and loyalty and increase turnover intention. Fischmann et al. (2018) reported that in Romania, low-income blue-collar workers and low-status white-collar workers would improve their performance when faced with job insecurity. Hu et al. (2019) proposed that when Chinese college students are confronted with negative feedback about their goals, those with low SES are more unlikely to goal separation and reduce goal adherence. As such, we propose:

*Hypothesis 3:* SES will negatively moderate the relationship between job insecurity and proactive behaviors. Specifically, the negative relationship between job insecurity and proactive behaviors will be weaker (vs. stronger) at low (vs. high) of employee’s SES.

### Three-Way Interactive Effect

In organizations, employee behaviors are inevitably influenced by both psychological and contextual variables which are simultaneously important elements influencing the path of self-control efforts (Baumeister and Vohs, 2016). Nevertheless, few studies have verified the jointly moderated effect of these factors on employee self-control in uncertain environments. Therefore, it is necessary to further explore the three-way interactive role of FWSS and SES in the relationship between job insecurity and employees’ different-oriented proactive behaviors in this study.

First, although we previously argue that high SES may decrease an employee’s self-control efforts on proactive behavior while experiencing job insecurity, it is interesting to note that motivation may alter this result. On the one hand, employees with high FWSS and high SES, not only possess sufficient material and social resources but also have more commitment to their self-set goals that are related to the activated possible work identity. Thus, when experiencing job insecurity, these employees will devote more attention and self-control effort in adhering to goals and responding to threats (Hu et al., 2019). The possible reasons are that they are less likely to experience dilemmas of proactivity in uncertainty and that this strategy would facilitate access to acquisition of personal interests and the satisfaction of psychological needs, such as competence and achievement. On the other hand, a prior study suggests that individuals with low SES are more likely to be exposed to environmental threats and social injustice; thus, their self-control strategies are more connected to beliefs regarding the relationship between the present behavior and future success (Laurin et al., 2011; Hu et al., 2020). Specifically, clear goals and the belief that they can achieve them through their own efforts (high FWSS) will compensate for their early failure and motivate them to apply their self-control resources in pursuit of the long-term goals. In sum, consistent with hypotheses 1 and 2, this study proposes that FWSS positively moderates the relationship between job insecurity and proactive skill development, individual task proactivity among employees of different SES.

Second, for employees with low FWSS and high SES, job insecurity may be interpreted as a barrier to maintaining personal interests and values in the short-term, due to the availability of preferable opportunities and the absence of clear future career goals. Especially when the threat and uncertainty created by job insecurity cannot be easily changed, they are more likely to focus on how to restore personal control in the short-term through the advantageous resources available to them rather than through the proactivity at work and career. Conversely, for employees with low FWSS and low SES, although higher economic and emotional vulnerability may stimulate the employees’ desire to protect available resources or meet employer expectations, the lack of motivation makes it difficult for them to choose from the dilemma of proactive behavior. As a result, they are more likely to make efforts to improve occupational skills and improve task performance in order to protect their current jobs. Considering the arguments above, we hypothesize:

*Hypothesis 4:* The FWSS and SES will have a positive three-way interactive effect on the relationship between job insecurity and individual-oriented proactive behavior.

## Materials and Methods

### Participants and Procedures

We conducted a two-wave survey study. The data was collected from 10 Chinese companies in Jiangsu and Hunan provinces. With the assistance of the human resources managers, we randomly distributed questionnaires to employees and asked them to complete them carefully. The participants were voluntary. In the beginning, all participants were informed about the academic research use of the survey data and ensured full confidentiality of their responses, so as to receive honest responses. In the first-wave survey, we sent questionnaires to 340 employees and received 279 usable responses (response rate of 82.0%). The participants were required to report demographic variables (age, gender, education level, and tenure), and their perceptions of job insecurity, FWSS, and SES. One month later, in the second-wave survey, 279 employees were further asked to provide information on their proactive behavior (involving proactive skill development, individual task proactivity, and organizational member proactivity) and information for matching. In the second-wave survey, 227 responses were finally matched and were valid (response rate of 66.8%). Among the final sample, 48.5% were male and 51.5% were female; the mean age was 30.110 years (*SD* = 5.573); the education level was mainly concentrated on undergraduate degrees, accounting for 74.9%: the average tenure (in years) was 4.357 years (*SD* = 4.357).

### Measures

We created a Chinese version for all variables in our survey following the translation-back-translation procedures. Unless otherwise specified, the variables were measured on the same 5-point Likert scale, ranging from 1 (strongly disagree) to 5 (strongly agree).

#### Job Insecurity

A three-item scale developed by Hellgren and Sverke (2003) was used. A sample item was “I am worried about having to leave my job before I would like to.” Cronbach’s alpha was 0.868.

#### Future Work Self Salience

We measured the FWSS using the simplified three-item scale (in Study 1b) used by Strauss et al. (2012). A sample item was: “The mental picture of this future is very clear.” Before completing the questionnaire, the participants were asked to “mentally travel into the future and to imagine the future work self you hoped to become, and keep this mental image in mind.” Cronbach’s alpha was 0.860.

#### Socioeconomic Status

Following Adler et al. (2000), we measured participant perceptions of their SES using a single item. We showed the participants a picture of a 10-rung ladder and informed them that, “Think of this ladder as representing where people stand in our society. At the top of the ladder (number 10) are the people who are the most advantaged in terms of money, education, and employment. At the bottom (number 1) are the people who are the most disadvantaged.” They were then asked to choose their current position from the numbers 1–10.

#### Proactive Behavior

Organizational member proactivity and individual task proactivity were both measured using the three-item scale developed by Griffin et al. (2007); proactive skill development was measured using the three-item scale developed by Claes and Ruiz-Quintanilla (1998). Participants were asked to respond to how frequently they were engaged in the following behavior over the last several weeks. The sample items are as follows: “made suggestions to improve the overall effectiveness of the organization,” “initiated better ways of doing your core tasks,” and “developed skills which may be needed in the future.” The response scale ranged from “much less than usual” (1) to “much more than usual” (5). Cronbach’s alpha for each of the three scales was 0.841, 0.825, and 0.822.

#### Control Variables

In line with a previous study, four demographic variables were controlled in the present study including gender, age, education, and tenure (Strauss et al., 2017; Xu et al., 2021). Gender (0 = men, 1 = women) and education (1 = junior college and below, 2 = undergraduate college, 3 = postgraduate and above) were dummy-coded. Age and tenure were measured in the number of years.

## Results

### Descriptive Statistics Analysis

The mean, SD, and correlation coefficients of the variables are present in Table 1. The results show that there is a significant negative correlation between job insecurity and organizational member proactivity, individual task proactivity, and proactive skill development. In addition, FWSS and SES positively relate to all the types of proactive behavior.

**TABLE 1:** Correlations, means, SDs, AVEs, and CR among the variables.

Variables	Mean	*SD*	1	2	3	4	5	6	7	8	9	10	CR
(1) Gender	0.515	0.501											
(2) Age	30.110	5.573	−0.144*										
(3) Education	1.943	0.499	–0.041	–0.047									
(4) Tenure (in years)	4.357	3.611	–0.090	0.603**	–0.079								
(5) JI	2.438	0.979	0.070	–0.070	–0.015	–0.117	(0.834)						0.872
(6) OMP	3.705	0.774	−0.142*	0.073	0.025	0.082	−0.247**	(0.801)					0.842
(7) ITP	4.009	0.655	–0.072	0.056	0.024	0.073	−0.311**	0.647**	(0.782)				0.825
(8) PSD	4.073	0.651	–0.058	–0.062	0.113	–0.013	−0.302**	0.579**	0.660**	(0.798)			0.837
(9) FWSS	3.794	0.741	0.009	0.022	0.064	0.005	−0.182**	0.312**	0.420**	0.436**	(0.821)		0.861
(10) SES	5.678	1.582	0.043	0.129	0.150*	0.235**	−0.264**	0.178**	0.185**	0.139*	0.197**	/	/

*JI, job insecurity; OMP, organizational member proactivity; ITP, individual task proactivity; PSD, proactive skill development.*

*The square root of AVE is in parentheses on the diagonal.*

**p < 0.01; *p < 0.05.

### Confirmatory Factor Analysis

In order to analyze the distinctiveness of the study variables before the hypothesis test, we conducted a confirmatory factor analysis (CFA) on the five constructs of job insecurity, FWSS, organizational member proactivity, individual task proactivity, and proactive skill development using AMOS 20.0. Both Tables 1, 2 show the results of the CFA of our study variables. The model fit of the hypothesized five-factor model (χ^2^/*df* = 1.405, *CFI* = 0.982, *TLI* = 0.977, *IFI* = 0.983, *GFI* = 0.939, *RMSEA* = 0.042) was acceptable. We then compared the hypothesized five-factor model with four alternative models. The Chi-square difference results show that the hypothesized five-factor model is significantly better than the four-factor model (displayed in Table 2). It is indicated that our measurement has well discriminant validity. In addition, based on the hypothesized model, we calculated the CR and AVE for each variable which is shown in Table 1, and the results also supported the discriminant validity and construct validity for each variable.

**TABLE 2:** Confirmatory factor analysis (CFA).

Model	χ^2^	*df*	χ^2^/*df*	*CFI*	*TLI*	*IFI*	*GFI*	*RMSEA*
Five-factor model (A, B, C, D, E)	112.374	80	1.405	0.982	0.977	0.983	0.939	0.042
Four-factor model (A + B, C, D, E)	451.121	84	5.370	0.800	0.750	0.802	0.766	0.139
Three-factor model (A + B + C, D, E)	681.404	87	7.832	0.676	0.609	0.680	0.686	0.174
Two-factor model (A + B + C + D, E)	716.936	89	8.055	0.658	0.597	0.661	0.684	0.177
Single-factor model (A + B + C + D + E)	827.321	90	9.192	0.599	0.532	0.602	0.667	0.190

*A, JI; B, FWSS; C, OMP; D, ITP; E, PSD.*

Since all variables were answered by employees, a common method bias test was still required. Harman’s single-factor test indicated that in the unrotated principal component analysis of 5 five factors, the largest factor explained 39.117% of the variance, less than 50%. The unmeasured latent method factor technique was used to further test for common method bias. Especially, in the hypothesized seven-factor model, an uncorrelated method factor was added which connected all the items. The results show that the method effect model is not significantly better than the hypothesized model (the difference of all Fit index range from 0.01 to 0.02). Thus, it could be considered that our findings are unlikely to be seriously affected by the common method variance.

### Hypothesis Test

We used multiple regression analysis to test our hypothesis. Hypothesis 1 predicted that job insecurity would have a differential negative effect on different types of proactive behaviors. We included control variables in Model 1, Model 4, and Model 7 (i.e., one dummy variable for gender, age, education, and tenure), and added the respective main effects (job insecurity). As shown in Table 3, in Models 1, 4, and 7, job insecurity was a negative significant for organizational member proactivity (OMP (β = –0.233, *p* < 0.001), individual task proactivity (ITP) (β = –0.304, *p* < 0.001) and proactive skill development (PSD) (β = –0.303, *p* < 0.001). However, there is a non-difference between the negative relationships. Hypothesis 2 predicted that FWSS would attenuate the negative relationship between job insecurity and proactive behavior and the moderating effect would be stronger on ITP and PSD. According to the recommendations of Aiken and West (1991), we further added FWSS and the interaction term in Models 1, 4, and 7 respectively. Models 2, 5, and 8 in Table 3 showed that the two-way interaction term of job insecurity and FWSS is positively significant. Statistically, the coefficient of OMP (β = 0.138, *p* < 0.05) is weaker than ITP (β = 0.242, *p* < 0.001) and closer to PSD (β = 0.146, *p* < 0.01). Hypothesis 3 predicted that SES would increase the negative relationship between job insecurity and proactive behavior. We added SES and the interaction term in Models 1, 4, and 7 respectively. In Models 3, 6, and l 9 of Table 3, the two-way interaction term of job insecurity and SES is negatively significant for OMP (β = –0.172, *p* < 0.01), ITP (β = –0.192, *p* < 0.01), and PSD (β = –0.168, *p* < 0.01).

**TABLE 3:** Hierarchical regression results of two-way interactive role on proactive behavior.

Variables	OMP			ITP			PSD		
	Model 1	Model 2	Model 3	Model 4	Model 5	Model 6	Model 7	Model 8	Model 9
Gender	–0.238	–0.230	–0.285	–0.092	–0.071	–0.139	–0.089	–0.085	–0.124
Age	0.004	0.002	0.009	0.002	0.000	0.009	–0.016	–0.019	–0.011
Education	0.040	0.011	0.004	0.041	0.004	0.010	0.207	0.164	0.191
Tenure (in years)	0.009	0.010	–0.003	0.008	0.009	–0.005	0.003	0.005	–0.006
JI	−0.233***	−0.180**	−0.210**	−0.304***	−0.231***	−0.284***	−0.303***	−0.229***	−0.294***
FWSS		0.281***			0.380***			0.393***	
SES			0.143*			0.131^†^			0.080
JI × FWSS		0.138*			0.242***			0.146**	
JI × SES			−0.172**			−0.192**			−0.168**
*F*	3.811**	6.756***	4.639***	4.965***	14.025***	5.823***	5.526***	12.451***	5.476***
*R-squire*	0.079	0.178	0.129	0.101	0.310	0.157	0.111	0.285	0.149
Δ*R-squire*		0.099	0.050		0.209	0.056		0.174	0.038

^†^p < 0.1, *p < 0.05, **p < 0.01, ***p < 0.001.

*All predictors were centered.*

**FIGURE 2:**
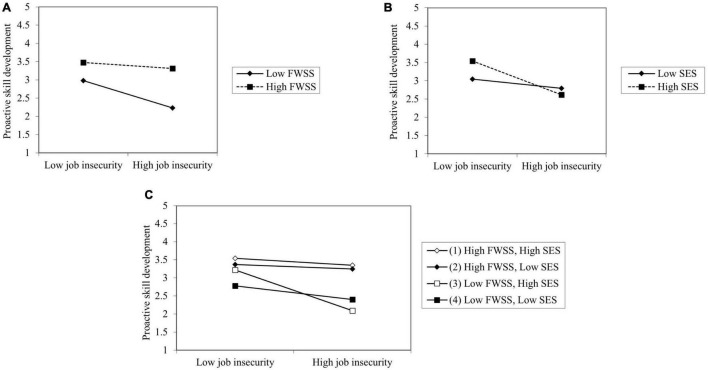
The moderating role of FWSS and SES on the relationship between job insecurity and PSD. **(A,B)** Two-way interaction. **(C)** Three-way interaction.

**FIGURE 3:**
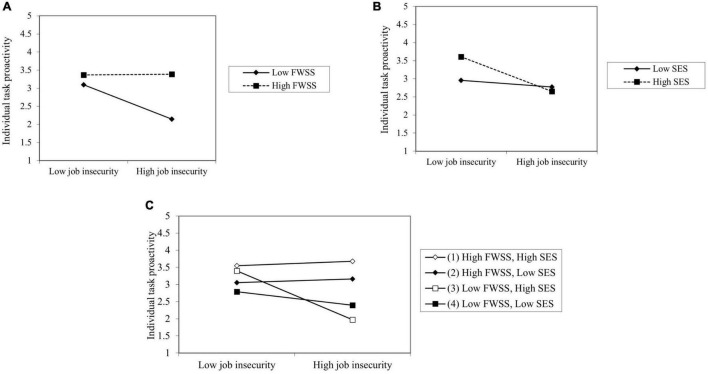
The moderating role of FWSS and SES on the relationship between job insecurity and ITP. **(A,B)** Two-way interaction. **(C)** Three-way interaction.

Hypothesis 4 predicted that job insecurity, FWSS, and SES would have a three-way interactive effect on proactive behavior and the effect would be stronger on ITP and PSD than on OMP. We successively added all variables in Hypotheses 1–3, two-way interaction terms of FWSS and SES, three-way interaction terms of job insecurity, FWSS, and SES in Models 10, 11, and 12. Model 11 of Table 4 showed that the two-way interaction term of FWSS and SES (β = 0.102, *p* < 0.05) and three-way interaction term (β = 0.133, *p* < 0.01) is positively significant. However, in Model 10 neither of these terms were significant; in Model 12, the three-way interaction term (β = 0.084, *p* < 0.1) was slightly positively significant. As is shown in Figures 2, 3, we followed the procedures of Dawson and Richter (2006) and plotted the slope analysis for two-way and three-way interactions. Thus, Hypotheses 2–4 were supported, Hypothesis 1 was partially supported.

**TABLE 4:** Hierarchical regression results of three-way interactive role on proactive behavior.

Variables	OMP	ITP	PSD
	Model 10	Model 11	Model 12
Gender	–0.268	–0.116	–0.110
Age	0.006	0.008	–0.015
Education	–0.032	–0.025	0.150
Tenure (in years)	0.000	–0.004	0.000
JI	−0.174**	−0.198**	−0.229***
FWSS	0.241***	0.362***	0.377***
SES	0.125^†^	0.150*	0.050
JI × FWSS	0.150**	0.258***	0.149**
JI × SES	−0.130*	−0.126*	−0.102^†^
FWSS × SES	–0.022	0.102*	0.018
JI × FWSS × SES	0.063	0.133**	0.084^†^
*F*	5.429***	11.933***	8.847***
R*-squire*	0.217	0.379	0.312
ΔR*-squire*	0.138	0.278	0.201

^†^p < 0.1, *p < 0.05, **p < 0.01, ***p < 0.001.

*All predictors were centered.*

## Discussion

Drawing upon the strength model of self-control, our study investigated and compared the effects of job insecurity on different types (individual-oriented and organization-oriented) of proactive behaviors, and the moderating role of FWSS and SES for this relationship. As predicted, we found that job insecurity had a negative effect on all proactive behaviors. The FWSS attenuated the effect of job insecurity on all types of proactive behaviors (the strongest on ITP), while SES strengthened the relationship. In addition, FWSS and SES play a three-way interacted role in the relationship between job insecurity and proactive behaviors (specifically ITP and PSD).

### Theoretical Implications

The present study makes several contributions to the literature. First, we enriched the theoretical perspective on the mechanism of how job insecurity influences proactive behavior and explored the potential differences in the antecedents of different-oriented proactive behaviors. On the one hand, most prior studies are based on the perspective of social exchange theory and on individual motivation to explore how an individual responds to job insecurity and why he is engaged in proactivity (Lam et al., 2015; Xu et al., 2021). In the present study, we identified job insecurity as uncertainty and theoretically reiterate that both coping strategies for the negative outcomes of job insecurity and acting in proactivity are resource-intensive (Selenko et al., 2017; Strauss et al., 2017). This suggests that the strength model of self-control can provide a further theoretical basis for clarifying the mixed finding regarding the relationship between job insecurity and the proactive behavior of an employee. On the other hand, most of the existing studies do not distinguish the different types of proactive behaviors and compare their antecedent. Although our findings supported the idea that job insecurity has a direct negative effect on different types of proactive behaviors (Wang et al., 2019; Yao et al., 2021), we likewise found that this outcome varies across some boundary conditions which can alter an individuals’ self-control effort. This provides partial empirical evidence for a classification study of proactive behavior.

Second, we respectively examined the moderating effects of FWSS and SES on job insecurity and different-oriented proactive behaviors. These findings shed light on the fact that in the case of self-control failure, whether employees maintain a high level of self-control in subsequent actions not only depends on motivation but is also influenced by contextual factors. On the one hand, we simultaneously considered individual- and organization-oriented proactive behaviors and found that in experiencing job insecurity, the motivation provided by FWSS was more likely to make ITP and PSD a way to deal with job insecurity. This response to the call of Shoss (2017) and Cai et al. (2019) to enrich the study of employees’ job preservation strategies in experiencing job insecurity and provides a new perspective for future research on the antecedents of proactive behavior. On the other hand, we also found that individuals with low SES may apply more self-control effort to protecting existing resources and keep consistent with the expectations of others due to multifaceted vulnerability. These results support the propositions that SES can make employees attach different values to personal interests or expectations of others, and even change the direction of the use of self-control resources (Flores et al., 2017). This goes beyond Berntson et al. (2010) and indicates that a shortage of such objective resources can influence and limit individual strategies for coping with uncertainty and threats.

Third, we contribute to the strength model of self-control and SES literature. We found that FWSS and SES have a three-way interaction on the relationship between job insecurity and ITP and PSD. Previous studies have primarily examined the moderating role of motivation on self-control failure (Baumeister and Vohs, 2016). Our study extends the idea and argues that motivation can change the effect of objective conditions on self-control. Moreover, it is intriguing to argue that beliefs to the future can change the tactics of individuals with high SES in their facing of threats, and apply more self-control resources to acquire resources in the future, even when they are at risk of losing them. This provides new evidence for the mechanisms that shape the coping strategies of (dis)advantage for employees under adversity.

### Practical Implications

Our study has several practical implications in terms of intervention strategies in the negative outcomes of job insecurity. Nowadays, job insecurity is becoming an inevitable threat. Although a certain degree of the negative environment may promote employees’ proactive behavior, if there is a lack of intervention, job insecurity would have a great harmful impact on both the company and the employees. In our study, we found that employees’ self-initiated goals based on a clear and positive work-related self can effectively interfere with the negative effect of job insecurity on proactive behavior, and the higher are the employees with SES, the stronger is the intervention effect.

Accordingly, first, organizations and managers should pay more attention to the construction of the employees’ future work self. They should change the traditional way of performance management and build a performance appraisal system that combines employee evaluation and development that can guide the employees to clarify their future work goals and career growth in their daily work life. It is especially critical for newcomers who have a strong need for achievement. Moreover, improving the training system and career planning guidance is also necessary. Since it contributes employees to making the multiple future selves, it includes more elements of organizational member identity and set goals that are aligned with the organization. Hence, employees act in ways that benefit both their personal goals and organizational development even in negative situations.

Second, it is necessary to pay attention to employees with different SES. On the one hand, although people with low SES are more susceptible to threats, it is undeniable that they will also worry about the loss of scarce resources and focus on conservation of resources which results in behaviors consistent with the organizational expectations. Therefore, in addition to helping them build a clear future working self, organizations also should provide clear expectations and fair treatment in the process of performance management and training, so that they can apply self-control resources to realize organizational expectations and beliefs of future achievements. On the other hand, for people with high SES, the organization and managers should guide them to apply rich social resources to their current work and establish goals consistent with the long-term development of employees and the organization.

### Limitations and Directions for Future Research

Although our study has some theoretical contributions to literature, several limitations also should be noted. First, since all our data are derived from employee self-reports, there is a risk of common method bias. Some means of *ex ante* control are adopted to reduce the effects of common method bias, for instance, conducting a two-way survey and emphasizing to the participants that all their responses would be kept confidential and used for academic research purposes. Meanwhile, *post hoc* tests on data, such as Harman single factor test, unmeasured latent method factor technique, and the significant interaction effect also indicate that our results are less likely to be seriously influenced by common method bias. Nevertheless, we still cannot directly infer the causal relationship between the variables. Future research can further investigate the possible mechanisms of why employees act in proactivity in negative contexts by means of event systems theory, field experiments, or qualitative research.

Second, we only investigated the moderating role of FWSS and SES, future research needs to further explore the potential mediating mechanisms between uncertainty and proactive behavior on the basis of this study. Although scholars have begun to explore the influencing mechanism of job insecurity from different perspectives, such as social identity theory, it can be seen from the results including our study that the positive effects of job insecurity may be “masked” by the strong negative effects. Therefore, future research can further explore the facilitating conditions of positive and inhibiting conditions of negative mechanisms of job insecurity. We encourage future research to further uncover the moderators of positive and negative job insecurity outcomes in different contexts.

## Conclusion

With the impact of uncertainty intensification, proactive behavior is becoming one of the indispensable determinants for organizational and individual success in uncertainty. Building on the strength model of self-control, this study examines how and when uncertain factors affect employees’ different types of proactive behaviors. On the one hand, seen from the perspective of self-control, we theoretically reiterate the resources-intensive nature of acting in proactivity and find that job insecurity has a negative effect on all types of proactive behaviors. On the other hand, given the multiple sources of job insecurity (Keim et al., 2014), completely eliminating job insecurity is infeasible for organizations and managers. Therefore, it is necessary to explore the factors that can mitigate the negative effect of job insecurity on proactive behavior. Although past studies have made considerable efforts in this area (Jiang, 2018), results of this study reveal that FWSS and SES, as motivational and situational factors that affect employee self-control respectively, can independently and interactively moderate the relationship between job insecurity and proactive behavior (particularly the individual-oriented proactive behaviors). These findings not only extend the strength model of self-control, but also provide a reference for managers to develop differentiated management strategies for different employees.

## Data Availability Statement

The original contributions presented in the study are included in the article/supplementary material, further inquiries can be directed to the corresponding author/s.

## Author Contributions

KH: research design, conception, original draft, data analysis, and critical revising for important intellectual content. JW: critical revising of important intellectual content, funding acquisition, and project administration. MS: original draft, data analysis, and critical revising of important intellectual content. All authors: contributed to the article and approved the submitted version.

## Conflict of Interest

The authors declare that the research was conducted in the absence of any commercial or financial relationships that could be construed as a potential conflict of interest.

## Publisher’s Note

All claims expressed in this article are solely those of the authors and do not necessarily represent those of their affiliated organizations, or those of the publisher, the editors and the reviewers. Any product that may be evaluated in this article, or claim that may be made by its manufacturer, is not guaranteed or endorsed by the publisher.
